# Time-reversed wave mixing in nonlinear optics

**DOI:** 10.1038/srep03245

**Published:** 2013-11-19

**Authors:** Yuanlin Zheng, Huaijin Ren, Wenjie Wan, Xianfeng Chen

**Affiliations:** 1 Department of Physics, Key Laboratory for Laser Plasmas (Ministry of Education), Shanghai Jiao Tong University, Shanghai 200240, China; 2 University of Michigan-Shanghai Jiao Tong University Joint Institute, Shanghai Jiao Tong University, Shanghai 200240, China

## Abstract

Time-reversal symmetry is important to optics. Optical processes can run in a forward or backward direction through time when such symmetry is preserved. In linear optics, a time-reversed process of laser emission can enable total absorption of coherent light fields inside an optical cavity of loss by time-reversing the original gain medium. Nonlinearity, however, can often destroy such symmetry in nonlinear optics, making it difficult to study time-reversal symmetry with nonlinear optical wave mixings. Here we demonstrate time-reversed wave mixings for optical second harmonic generation (SHG) and optical parametric amplification (OPA) by exploring this well-known but underappreciated symmetry in nonlinear optics. This allows us to observe the annihilation of coherent beams. Our study offers new avenues for flexible control in nonlinear optics and has potential applications in efficient wavelength conversion, all-optical computing.

Time-reversal symmetry dominates in many physical systems; it allows a physical process to reverse in a backward direction of time. This powerful symmetry enables many practical applications under such reversible principle, with examples including acoustic and electromagnetic focusing using time-reversal mirror^1^,^2^,^3^,^4^, phase conjugation mirror in optics^5^,^6^, etc. Especially in optics, light transmission in random medium can be enhanced by several orders by exploring the time-reversal symmetry^7^,^8^,^9^, similarly for enhancing second harmonic signals in random medium^10^. Recently, a novel concept named “coherent perfect absorber” (CPA)^11^,^12^,^13^ that explores time-reversed process of laser emission has shown that incident coherent optical fields can be perfectly absorbed by a time-reversed optical cavity, where the gain is replaced with an equal amount of loss. Also, the incident fields and frequency should coincide with those of corresponding lasing modes with gain under time symmetry. Recent efforts have studied CPA properties with different geometries^11^,^12^,^13^,^14^,^15^,^16^,^17^,^18^, though most in the linear optics regime. A nonlinear version of CPA has been theoretically proposed to investigate signal and idler beams' phase-varying dynamics in the presence of a pumping beam under a time-reversed optical parametric oscillation (OPO) scheme^14^. These studies of time symmetry have been attracting increasing attention, since they provide alternative and substantial ways to manipulate light in the nonlinear regime.

In this work, we experimentally exam the time-reversal symmetry for two classical nonlinear wave-mixing processes–SHG and OPA, characterize their nonlinear properties as opposite to their time-reversal counterparts, and reveal the nontrivial dynamics of phase varying in time-reversed nonlinear wave-mixing schemes. These backward-nonlinear wave mixings lead to the unique property of annihilation of coherent beams in a nonlinear quadratic medium by time reversal. Unlike the case of CPA in the linear regime where incident fields are totally absorbed and converted into heat^11^,^14^, annihilation of incident fields can lead to the generation of new fields. Such backward-parametric interactions may have a future application in efficient wavelength conversion for better long-wavelength detection, e.g. mid-IR, THz. More interestingly, a flexible phase control can be utilized to probe nonlinear dynamics during wave mixing, and redirect wave mixing forward or backward in time. This offers new techniques for flexible control in nonlinear optics and has potential applications in all-optical computing.

## Results

### SHG and time-reversed SHG

**Figure 1:**
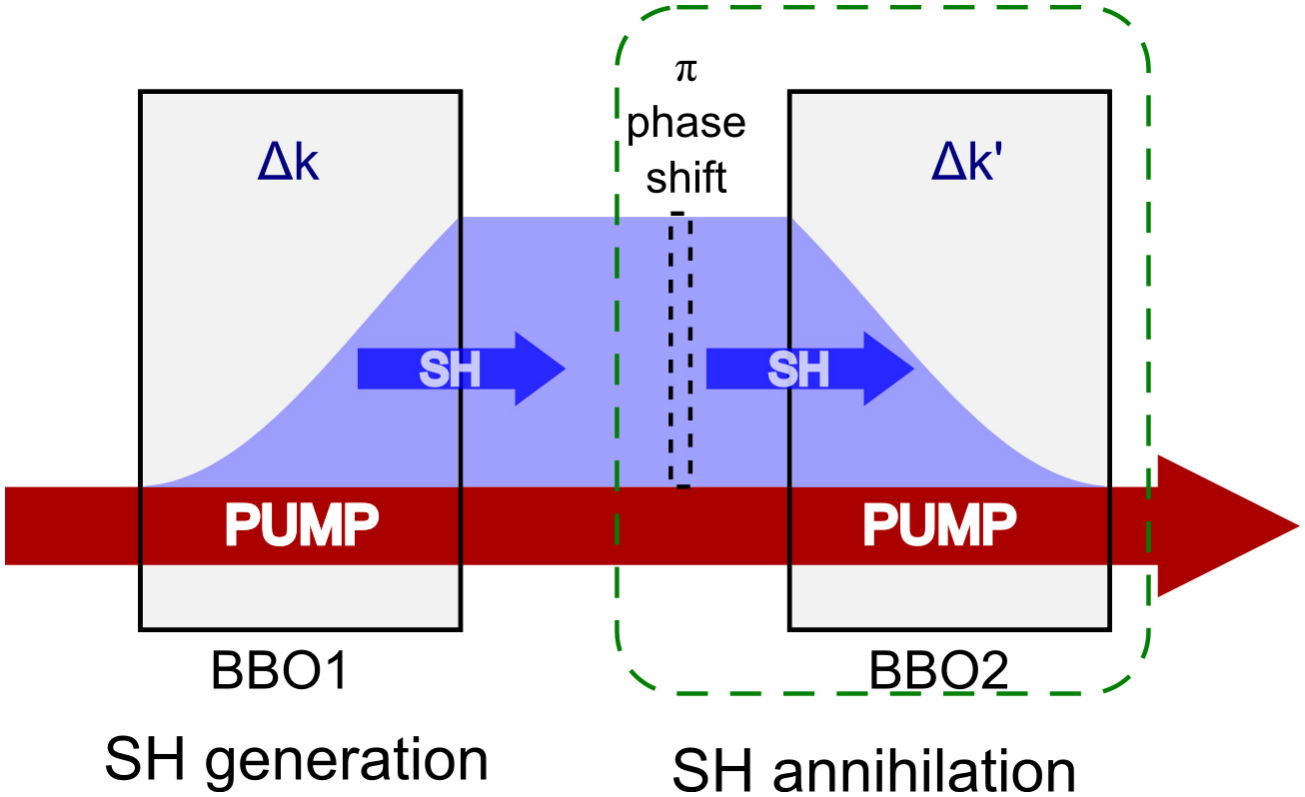
The schematic of SH generation and annihilation. The wave-mixing behavior of SHG process in the first nonlinear medium is time-reversed in the second (outlined in dashed box). The condition for time-reversal symmetry is obtained by adding a *π* phase shift to FW/SH phase difference and by flipping the sign of phase-mismatching vector (Δ*k*' = −Δ*k*). For a spontaneously grown SHG (starting from zero) in BBO1, its time-reversed process leads to “perfect” SH annihilation in BBO2 under time-reversal symmetry.

Figure 1 shows the scheme for time-reversed SHG. Two identical thin beta barium borate crystals (denoted BBO1 and BBO2 hereafter) cut for Type I SHG@1064 nm are used. The crystal length L is short enough (100 μm) to ensure pump non-depletion (small signal approximation), i.e. *A*_1_(*z*) = *A*_1_(0). The corresponding phase-mismatching vectors Δ*k* (Δ*k*') in BBO1 (BBO2) can be independently tuned by rotating the crystals. The intensity of the second harmonic (SH) after BBO1 is^21^: 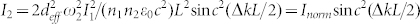, where *I* is the wave intensity. We refer the normalized SH intensity I_norm_ to unity hereafter, which equals the output of BBO1 at the phase-matching condition. *n_i_* is refractive index and *ε*_0_ is the vacuum permittivity. In BBO1, SH is generated as Δ*kL*/2 is controlled in the range of (−*π*, *π*), and the phase difference between the generated SH and FW is Δ*φ* = Δ*kL*/2. Thus BBO1 is both an SH source generator and a phase controller. Both the pump FW and generated SH are then incident into the second nonlinear medium--BBO2 after passing through a thin quartz plate for phase shift. For spontaneously-grown SHG (starting from zero) in BBO1, the time-reversed process points in exactly the opposite direction: SH signals with the appropriate relative amplitude and phase to the pumping beam can be totally annihilated when they incident onto a nonlinear crystal.

**Figure 2:**
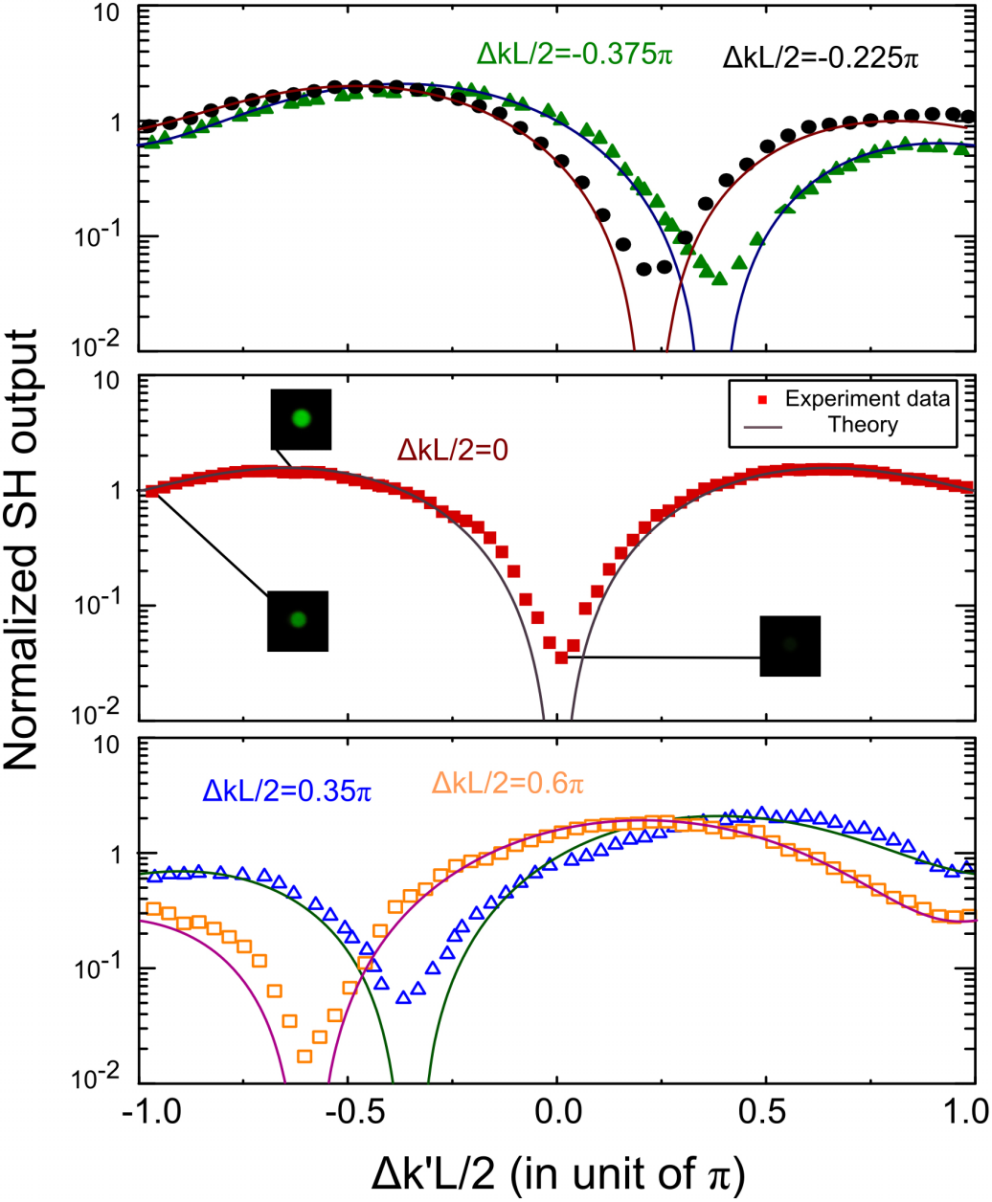
Experimental demonstration of reversed SHG. The measured total SH intensity with different FW/SH inputs into BBO2 produced by varying its phase-mismatching vector Δ*k*'. The dips are the signature of CPA, where SH generated in BBO1 is significantly absorbed by time-reversed SHG in BBO2. (Insets: Photographs of the SH spot at indicated conditions.).

We set BBO1 for several different conditions where Δ*kL*/2 = −0.375*π*, −0.225*π*, 0, 0.35*π*, 0.6*π*. Figure 2 shows the total SH output after BBO2 measured by scanning the phase mismatching vector Δ*k*'. Significant annihilation of SH in BBO2 occurs when time-reversal symmetry requirements are met, i.e. when the dips of the SH output curves are located at Δ*k*' = −Δ*k*. These results clearly indicate that, for a low conversion efficiency SHG, there always exists a symmetrically reversed SHG process that is capable of “perfect absorbing” its SH wave when coherently illuminated by both FW and SH. The corresponding attenuation of each dip of the curves is measured to be 11.5 dB, 12.4 dB, 14.5 dB, 11.4 dB and 10.6 dB, and yet the SH generated in BBO1 can, in theory, be totally absorbed in BBO2^11^,^14^. Such modulation depth is limited by experimental conditions, e.g. reflection, pumping intensity, wavefront, coherence^13^. Moreover, complete annihilation cannot be achieved if quantum noise is considered^14^,^24^.

### OPA and time-reversed OPA

**Figure 3:**
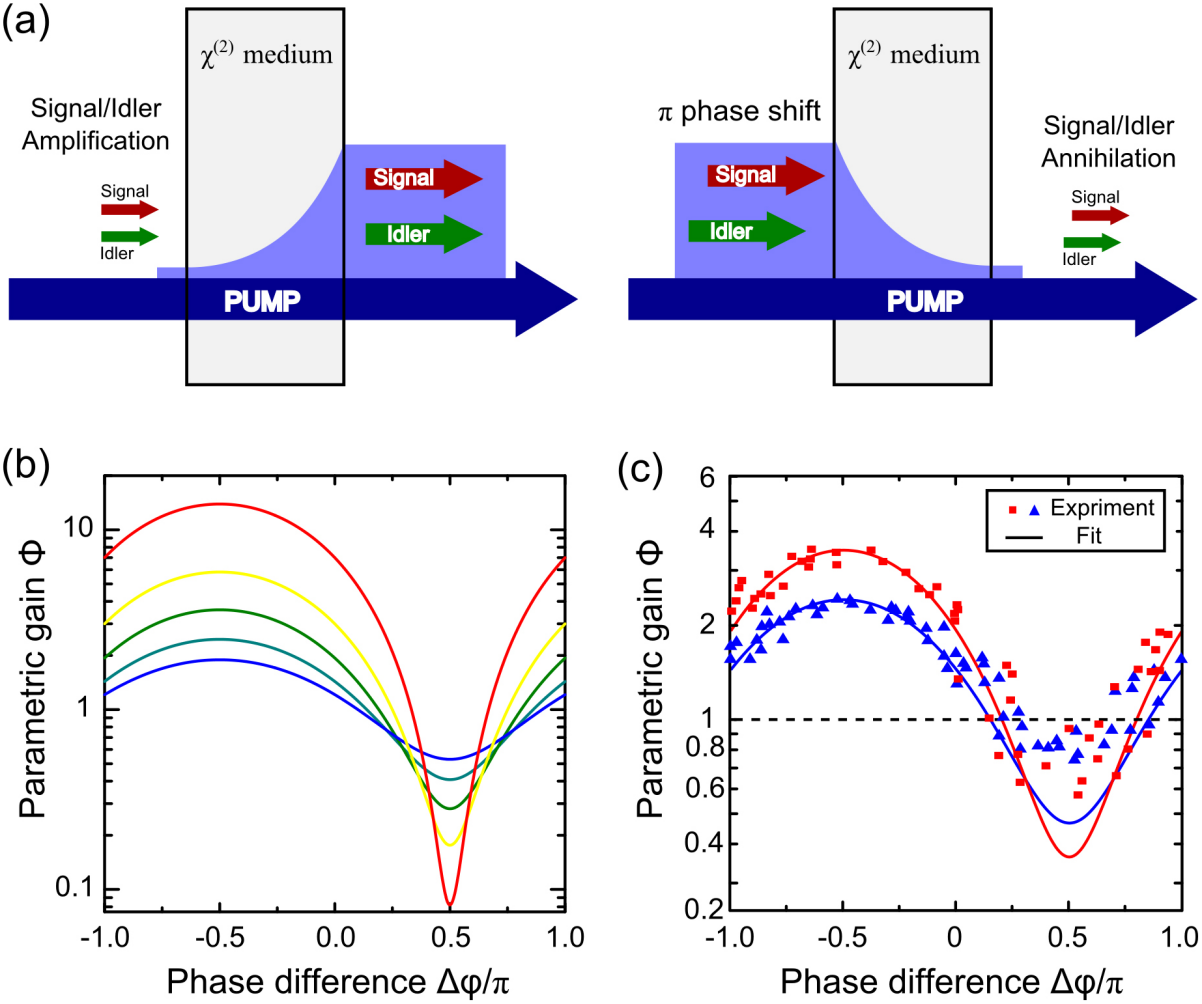
The schematic of OPA and its reversal. (a) The schematic of Signal/Idler pair amplification and annihilation. (b) Behavior of the parametric gain Φ vs. total phase difference Δ*φ* for various pump intensities. (c) The experimentally measured overall Φ vs. Δ*φ*. Gain variety along pump pulses is taken into account in data fitting.

OPA can be regarded as a reversed wave mixing to SHG from a quantum picture: OPA splits one photon into two while SHG combines two photons. However, they are not inherently time reversal to each other, for example, the initial pumping beams differ. Compared with reversed SHG, the pumping effect can play a critical role in reversed OPA geometry. Here we extend our study to a reversed two-channel OPA, which qualitatively requires two coherent input beams and a pumping beam. Annihilation of waves can only be achieved when the relative phase, amplitude and pumping level conditions are reached, as schematically shown in Fig. 3a.

In Fig. 3b and 3c, we show the theoretical and experimentally measured parametric gain when the phase difference between the three waves is varied. Far from Δ*φ* = *π*/2, the system behaves as an OPA device (Φ > 1). Note that at Δ*φ* = *π*/2, a dip with parametric gain Φ < 1 is observed in the gain curves, indicating Signal/Idler annihilation, when pumping above the threshold of OPA. Such a dip is the clear signature of “colored” OPA-CPA^14^, which is also the time-reversed process of its counterpart OPA above threshold. In an ideal case, the product of the gain factor for the in-phase and out-phase signal would be a unity. This is a straightforward outcome of time-symmetry rule. However, in our experiment, there are plenty of conditions that lead us to this result. One critical parameter is the pulse duration. The actual pulse of a pump wave with shorter time duration does not completely overlap with Signal/Idler pulses, which is the major reason for discrepancy between the experimental and theoretical curves, especially around the lowest attenuation points. Nevertheless, these dips still clearly indicate that a reversed-OPA device is capable of attenuating Signal/Idler given coherent illumination with correct phases^26^, which behaves as a “colored” OPA-CPA system^14^. The result demonstrates two operating states of a wave-mixing process in its forward and time-reversed direction. Nevertheless, in our experiment, the gain depletion regime is never reached. In this regime, OPA-CPA is still possible when energy can be drained completely from the pumping beam^14^,^27^. Here further study is required.

## Discussion

The SHG process converts a fundamental wave (FW) into its second harmonic (SH) through the nonlinear response of a medium. However, the energy flow can oscillate between the fundamental and harmonic waves, determined by the phase mismatch condition and phase difference of the interacting waves. In an undepleted-pump scheme, we consider the quasimonochromatic waves with carrier frequencies of fundamental wave at *ω*_1_, and second harmonic wave at *ω*_2_ = 2*ω*_1_. After representing the electric field as 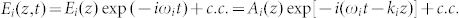, where *i* = 1, 2 refers to FW and SH, respectively, and where *A_i_*(*z*) is the slowly varying amplitude (slow-varying envelope approximation), the wave equation can be expressed as^21^: 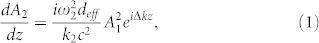
where the phase-mismatching vector is Δ*k* = 2*k*_1_ −*k*_2_ and *d_eff_* is the effective nonlinearity, and *c* is the speed of light. Generally, in the SHG process SH signals grow along the propagation in a nonlinear optical crystal. However, they may also decay due to the familiar problem of phase-matching. Here the phase plays an important role: it directs the time axis where energy flows. For example, for SHG schemes in quasi-phase-matching (QPM) gratings, one can purposely flip the 2nd-order susceptibility (*χ*^(2)^) of ferroelectric domains thus adding a phase jump to SH waves in order to prevent SH falling back to FW due to dephasing. This encourages SHG conversion. Fundamentally, this originates from an interference effect similar to the linear CPA, but such interference is nonlinear one due to nonlinear polarization between SH and FW beams at molecular level^19^. Thus, phase difference is crucial when considering the time-reversed process of SHG as well.

To consider the time-reversed counterpart, we take the complex conjugate of Eq. 1 to get 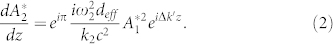
Note that Eq. 2 is the same as Eq. 1 except for an extra phase difference (Δ*φ* = *φ*_2_ − 2*φ*_1_ = *π*) and a sign-flipped phase-mismatching vector (Δ*k*' = −Δ*k*). The complex conjugate fields 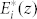, which represent the backward fields, satisfy Eq. 2. With such exact time-reversal configurations, SHG beams undergo a backward process with respect to the counterpart in the forward-time direction under a time reversible environment that excludes magnetism and loss. For a non-seeded scheme, SH starts to grow from quantum fluctuations draining the energy from FW along the propagation. The exact time reversal of the problem should lead SH to zero, as it is the initial stage for SHG, similar to CPA in the linear case. Here the model is based on a monochromatic wave or waves with simple envelopes. For more complex waves, one has to time-reverse waveforms in space and time as well, e.g. through time-reversed mirrors^22^ or nonlinear wave mixings^23^.

**Figure 4:**
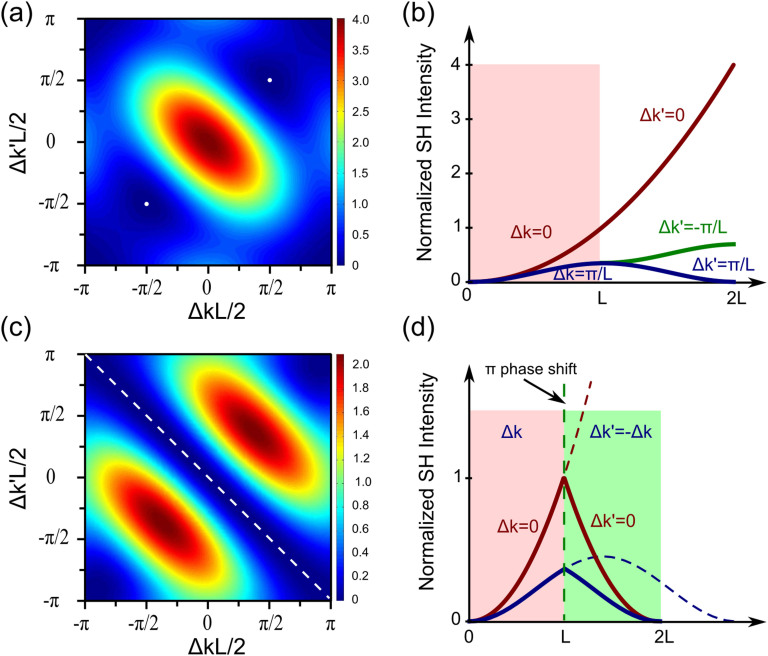
The total SH output characteristics and the SH intensity along the propagation path. (a) The normalized total SH output without a *π* phase shift between the two crystals. The two white dots show two situations when SH generated in BBO1 is cancelled out in BBO2 due to dephasing. (b) SH intensity along the propagation length in the two crystals in phase-matching and mismatching situations. (c) The normalized total SH output is greatly modified after introducing an additional *π* phase shift to Δ*φ* in between the two crystals. The diagonal dashed line shows total SH outputs equal to none, indicating any SH generated in the first nonlinear medium can always be cancelled out in the second. (d) SH intensity along the propagation length in the two crystals. The SH intensity evolution manifests itself in spatially symmetric pattern, which is also an indication of time-reversal of the wave mixing process. White dash dots and line in (a) and (c) indicate zero intensity.

To gain insight into the nonlinear wave-mixing properties of the proposed time-reversed structure, we calculate the total output after BBO2 by integrating Eq. 1 along the propagating path. The total SH output is studied by scanning phase-matching vectors for both crystals with/without a *π* phase shift to Δ*φ*, as shown in Fig. 4. The points of interest are where the SH generated in BBO1 is totally cancelled out in BBO2 leaving only FW out of BBO2. In Fig. 4a, where two crystals are simply cascaded (without phase shift), SH only vanishes if Δ*kL*/2 = Δk'*L*/2 = ±*π*/2 (The blank points at the four corners are not the desired ones, and this is due to non-SH input and the absence of SH generated by BBO2.). It is well-known that SHG at phase-mismatching condition induces energy flow oscillation between FW and SH at the period of twice of the coherent length *L_c_* = *π*/Δ*k*, one can also purposely manipulate SHG output by phase control^28^. However, in order to achieve the total absorption of SHG, we have to consider time-reversed scheme. Figure 4b shows the typical SH intensity along the propagating path at the conditions of phase-matched, quasi-phase-matched, and phase-mismatched conditions (red, green and blue lines, respectively). Note that SH only flows back under the phase mismatching condition, which occurs twice over the coherent length. And along its propagation, SH exhibits symmetry between the two crystals. This stimulates the quest to explore time-symmetry to re-converting SH back to FW at any moment despite their current phase relations.

According to the analysis above, time-reversal symmetry of SHG can be implemented by introducing an additional *π* phase difference before BBO2 and reversing the phase mismatching vector in BBO2. We re-plot the SH output after BBO2 in Fig. 4c. The SH generated in BBO1 can always be cancelled out in BBO2 whenever Δ*k*' = −Δ*k* is fulfilled. This reveals a condition where SHG generated by BBO1 undergoes annihilation along a time-reversed path in BBO2. Figure 4d illustrates this idea better by showing SH intensity along the propagating path across both crystals. With these conditions, the intensity profiles of SH preserves the spatial symmetry along the propagation axis through both crystals. Even more convincing is that this symmetry is preserved under the phase-matched condition in Fig. 4d. It is also worth to mention that the *π* phase jump is introduced purposely to trigger time-reversal symmetry using a tilt thin quartz plate sandwiched between two BBO crystals. In a nonlinear manner, such phase shift can arise nonlinearly in a “cascading nonlinearity”^29^, where an effective kerr-like χ^(2)^(3ω;2ω,ω) nonlinearity is created through cascading two χ^(2)^(2ω;ω,ω) processes inside one single second-order nonlinear crystal. However, in this geometry, time symmetry is not preserved; second harmonic beams are converted into third harmonics instead of the fundamental ones like in our case here. In all these circumstances, the phase plays a core role in determining the direction of the wave-mixing process.

Then we consider non-depleted Type II degenerate OPA of three quasimonochromatic waves with carrier frequencies of fundamental wave at *ω_p_*, signal and idler at *ω_s_* = *ω_i_* = *ω_p_*/2. By slow-varying envelope approximation, neglecting group velocity mismatch (GVM) and group velocity dispersion (GVD), the envelops satisfy the coupled wave equations^21^

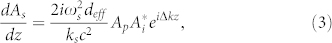


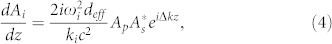
The subscripts *p*, *s*, *i* stand for pump, signal and idler, respectively. The phase mismatching vector Δ*k* = *k_p_* − *k_s_* − *k_i_* is required to be zero for an efficient OPA device. One can find the phase-matched parametric gain of signal/idler with respect to pump intensity and total phase difference (Δ*φ* = 2*φ_p_* − *φ_s_* − *φ_i_*) to be^14^,^21^,^25^: 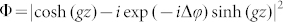, in which 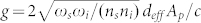 is proportional to the pump amplitude. The results with different pump intensities are plotted in Fig. 2b. Gain and attenuation are governed by the total relative phase Δ*φ*. Hence, the relative phase matters most when we consider the time-reversed OPA. Since Δ*k* = 0, OPA and its time-reversed process is accessible in the same condition, and an OPA device can behave like an OPA-CPA device^14^. Theoretically, the in-phase signal can be amplified by the factor Φ, whereas the out-of-phase signal is attenuated by the same factor. The dip in the gain curve at Δ*φ* = *π*/2 is a clear indicator of annihilation of Signal/Idler beams when pumping above the threshold of OPA.

In conclusion, we have demonstrated time-reversed wave mixings for SHG and OPA. This enables us to observe the annihilation of coherent beams under time-reversal symmetry by varying the relative phase of the incident fields. Time-reversed SHG is able to absorb coherent wave at second harmonic frequency of its pump. For time-reversed OPA, we show that the OPA can simultaneously amplify and attenuate coherent signal and idler waves when pumped above threshold. Our study provides a versatile platform for flexible control in nonlinear optics and potential applications in efficient wavelength conversion, all-optical computing.

## Methods

### Experiment setup for reversed SHG

The laser source used is an all-fiber femtosecond oscillator delivering 250 fs pulses centered at 1064 nm at a repetition rate of 80 MHz. A long-focal-length lens is used to loosely focus the light beam. And the two BBO crystals are placed on both side of the focus where the wavefront is also symmetrical. The desired *π* phase shift is obtained by inserting a tilt thin quartz plate in between. The tilt angle is calculated according to the dispersion relation such that SH wave accumulates an additional *π* phase shift than FW when passing through the plate.

### Experiment setup for reversed OPA

The gain media consist of a pair of anti-parallel BBO crystals pumped by an e-polarized frequency-doubled beam (532 nm) from a Q-switched nanosecond laser at a repetition rate of 20 Hz. The spatial wall-off of the three waves accumulated in the first crystal will be compensated by the second anti-parallel one. The crystals are cut for wavelength-degenerate type II OPA scheme pumped at 532 nm and are anti-reflection coated at both 532 and 1064 nm. The fundamental wave delivered by the laser is nominal 4 ns in duration (FWHM). And the pump beam, which is its second harmonic at 532 nm, has a nominal FWHM duration of 3 ns. A telescope system is used to reduce the pump beam diameter to 2 mm. The fundamental wave (1064 nm) is attenuated to the extant that is much weaker than the pump (<1/100), and its beam diameter is also reduced by a telescope system to a slightly smaller diameter to overlap with the pump. A half-wave plate is used to rotate the FW beam by 45 degrees to make the orthogonally polarized signal and idler input equal in amplitude on the onset of the crystal.

## Author Contributions

W.W. and X.C. designed and supervised the study; Y.Z. designed experiments setup, performed research, analyzed the data; Y.Z. and W.W. wrote the paper; H.R. provided advice and helpful theoretical discussion. All authors reviewed the manuscript.

## Supplementary Material

Supplementary Information Supplement
