# Supplementary material for: Time-reversed wave mixing in nonlinear optics
Source: Sci Rep. 2013 Nov 19;3:3245. doi: 10.1038/srep03245 (PMC3832849; doi:10.1038/srep03245)
Supplement: Supplementary Information — Supplement [file srep03245-s1.pdf]

Supplement for

Time-reversed wave mixing in nonlinear optics

Yuanlin Zheng, Huaijin Ren, Wenjie Wan* and Xianfeng Chen†
*,† To whom correspondence should be addressed.
E-mail: wenjie.wan@sjtu.edu.cn, xfchen@sjtu.edu.cn

1. Analysis for time-reversed SHG

Theoretic calculation of SH intensity

Considering the situation of SHG in two identical consecutive nonlinear media, the total amplitude of output SH is given in our case by integrating Eq. 1 in the manuscript from –L to L, and is simplified as

$$A_2 = \beta \cdot e^{-i\Delta kL}\left[\frac{1}{\Delta k}\left(e^{i\Delta kL}-1\right)+e^{i\Delta kL}\cdot\frac{1}{\Delta k'}\left(e^{i\Delta k'L}-1\right)\right],$$

with $\beta = \frac{\omega_2^2 d_{eff}}{k_2 c^2}$. The SH intensity is plotted in Fig. 2a in the manuscript.

For an exact time reversed SHG mixing, according to the analysis, one needs to add an additional π phase shift between the two waves, which corresponds to a minus sign adding to the first term in the square bracket. The overall SH output amplitude is

$$A_2 = \beta \cdot e^{-i\Delta kL}\left[-\frac{1}{\Delta k}\left(e^{i\Delta kL}-1\right)+e^{i\Delta kL}\cdot\frac{1}{\Delta k'}\left(e^{i\Delta k'L}-1\right)\right],$$

which is automatically cancelled out when $\Delta k' = -\Delta k$. The SH intensity is plotted in Fig. 2c in the manuscript.

Experimental method for time-reversed SHG

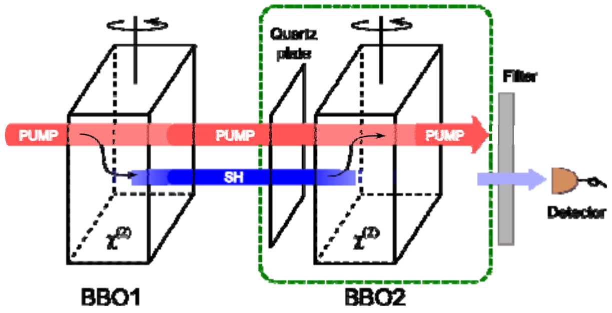

FIG. 1 | **The experimental setup for the demonstration of time-reversal SHG.** The reversed

SHG is outlined in the green dashed box.

In our experimental geometry, depicted in Fig. 1, two identical thin nonlinear crystals (BBO) are used, which were cut for Type I SHG@1064 nm. We first recorded the output of BBO1 in various incident angles, where the corresponding phase-mismatch vector and the phase difference of the output can be determined, as shown in Fig. 2a. The SH intensity at phase matching condition is normalized to unity. BBO1 is used SH source and fine phase controller for the input of the second BBO2. There are pros and cons of this scheme. One drawback is that to adjust the phase of the input onto BBO2, the intensity of SH is simultaneously varied. However, the advantage is that the appropriate SH-to-FW intensity ratio is always maintained for the second stage to demonstrate a "perfect" time-reversal SHG. In the demonstration of time-reversal SHG, a π phase shift between FW and SH was introduced by inserting a plate of quartz between the two crystals. Polarization of FW and SH is unaffected, but the dispersion induces different phase for each wave accumulated through the material. By proper alignment, the total phase difference $\Delta\varphi$ can be tuned to add another exact π shift on the onset on BBO2. The total SH output after BBO2 was recorded by a detector, the measured results are shown in Fig. 3 in the manuscript.

Here, the use of birefringence phase matching scheme is that it makes the tunability of phase matching vector rather convenient, not only its amplitude but also its sign, as depicted in Fig. 2b.

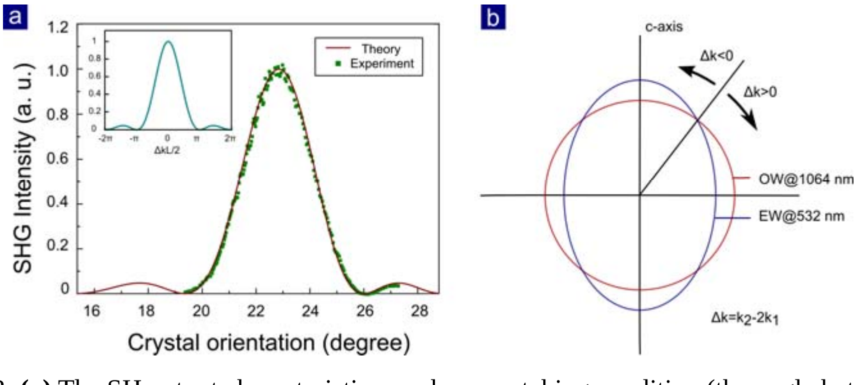

Figure 2: **(a)** The SH output characteristics as phase-matching condition (the angle between the wave propagation direction and optical axis) is changed. The thickness of the BBO crystal

is $100\ \mu m$. **(b)** The tunability of phase matching vector Δk through birefringence in a type I (oo-e) SHG scheme in negative birefringence crystal, BBO as is our experiment.

2. Experiment of OPA-CPA

Our experiment scheme to demonstrate OPA-CPA is depicted in Fig. 3. The system consists of a pair of anti-parallel BBO crystals, which are pumped by an e-polarized frequency-doubled beam (532 nm) from a Q-switched nanosecond laser at a repetition rate of 20 Hz. The crystals are cut for wavelength-degenerate type II OPA scheme pumped at 532 nm and are anti-reflection coated at both 532 and 1064 nm. The fundamental wave delivered by the laser is nominal 4 ns in duration (FWHM). And the pump beam, which is its second harmonic at 532 nm, has a nominal FWHM duration of 3 ns. A telescope system is used to reduce the pump beam diameter to 2 mm. The fundamental wave (1064 nm) is attenuated to the extant that is much weaker than the pump (<1/100), and its beam diameter is also reduced by a telescope system to a slightly smaller diameter to overlap with the pump. A half-wave plate is used to rotate the FW beam by 45 degrees to make the orthogonally polarized signal and idler input equal in amplitude on the onset of the crystal. Because of the critical wall-off nature of the birefringence phase matching and long crystal used in nanosecond OPA systems, the orthogonally polarized waves completely separate at the rear of the crystal, an identical gain medium oriented anti-parallel or direct backward reflection scheme is usually used to compensate the spatial walk-off.

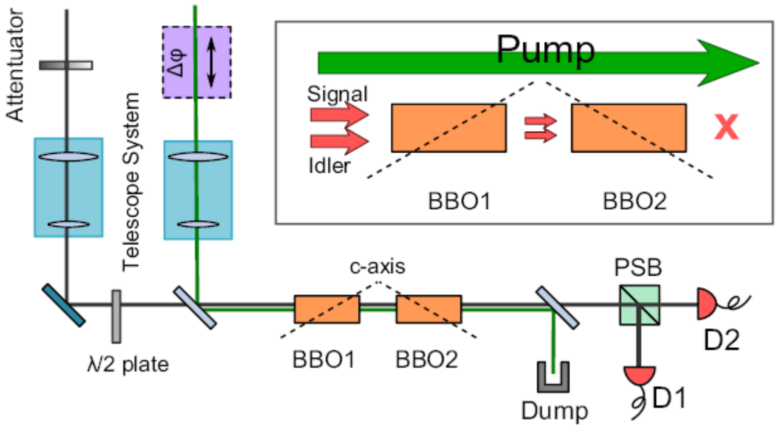

FIG. 3. **Schematic of reversed-OPA experiment.** The BBO crystals are cut for wavelength

degenerate Type-II OPA scheme, and their optical axes are placed anti-paralleled to compensate for spatial walk-off. The inset is a schematic of the CPA mechanism. The incident signal and idler beams with proper phase difference between the pump undergo de-amplification.

In our scheme, the spatial wall-off of the three waves accumulated in the first crystal will be compensated by the second anti-parallel one. The path lengths of the beam arms are made equal to within a few millimeters to ensure the time overlap. We adjust the phase of the pump pulses relative to the input Signal/Idler pulses by moving a corner cube prism in the pump beam path that is mounted upon a piezoelectric transducer (PZT). After the crystal, the pump beam is dumped and the output Signal/Idler beam is separated with a beam-splitting polarizer and detected with separate detectors. The experimentally measured parametric gain when the phase difference between the three waves is varied is shown in Fig. 4c in the manuscript.
